# Crystal structure and Hirshfeld surface analysis of ethyl 6′-amino-2′-(chloro­meth­yl)-5′-cyano-2-oxo-1,2-di­hydro­spiro­[indoline-3,4′-pyran]-3′-carboxyl­ate

**DOI:** 10.1107/S2056989021006459

**Published:** 2021-06-25

**Authors:** Farid N. Naghiyev, Maria M. Grishina, Victor N. Khrustalev, Mehmet Akkurt, Afet T. Huseynova, Anzurat A. Akobirshoeva, İbrahim G. Mamedov

**Affiliations:** a Department of Chemistry, Baku State University, Z. Khalilov str. 23, Az, 1148 Baku, Azerbaijan; b Peoples’ Friendship University of Russia (RUDN University), Miklukho-Maklay St. 6, Moscow, 117198, Russian Federation; c N. D. Zelinsky Institute of Organic Chemistry RAS, Leninsky Prosp. 47, Moscow, 119991, Russian Federation; d Department of Physics, Faculty of Sciences, Erciyes University, 38039 Kayseri, Turkey; e Acad Sci Republ Tadzhikistan, Kh Yu Yusufbekov Pamir Biol Inst, 1 Kholdorova St, Khorog 736002, Gbao, Tajikistan

**Keywords:** crystal structure, spiro­oxindole, dimers, hydrogen bond, Hirshfeld surface analysis

## Abstract

The mol­ecular conformation of the title compound, C_17_H_14_ClN_3_O_4_, is stabilized by an intra­molecular C—H⋯O contact, forming an *S*(6) ring motif. In the crystal, the mol­ecules are connected by N—H⋯O hydrogen-bond pairs along the *b-*axis direction as dimers with *R* ^2^_2_(8) and *R* ^2^_2_(14) ring motifs and as ribbons formed by inter­molecular C—H⋯N hydrogen bonds. There are weak van der Waals inter­actions between the ribbons. The most important contributions to the surface contacts are from H⋯H (34.9%), O⋯H/H⋯O (19.2%), C⋯H/H⋯C (11.9%), Cl⋯H/H⋯Cl (10.7%) and N⋯H/H⋯N (10.4%) inter­actions, as concluded from a Hirshfeld surface analysis.

## Abstract 2

The mol­ecules are connected in the crystal by N—H⋯O hydrogen-bond pairs along the *b-*axis direction as dimers with 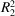
(8) and 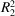
(14) ring motifs and as ribbons by inter­molecular C—H⋯N hydrogen bonds. Between the ribbons, there are weak van der Waals contacts.

## Chemical context   

**Figure 1:**
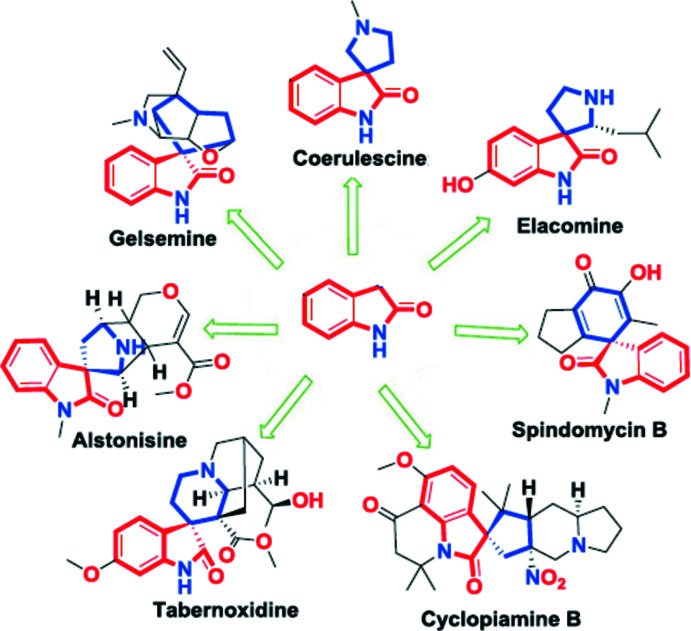
Natural products containing the spiro­oxindole motif.

Being the most significant tools in organic synthesis, carbon–carbon and carbon–heteroatom coupling reactions are important for the construction of fine chemicals such as pharmaceuticals, fragrances, anti­oxidants, *etc*. (Yadigarov *et al.*, 2009 ▸; Khalilov *et al.*, 2018**a* ▸,b* ▸; Zubkov *et al.*, 2018 ▸). These methods have found widespread application in the design of diverse heterocyclic ring systems, as well as spiro-heterocyclic compounds (Gurbanov *et al.*, 2018 ▸; Maharramov *et al.*, 2019 ▸; Mahmoudi *et al.*, 2019 ▸; Mamedov *et al.*, 2019 ▸; Yin *et al.*, 2020 ▸). The spiro­oxindole moiety is a key bioactive fragment of various natural products (Fig. 1 ▸), series of derivatives already being used in medicinal practice (Zhou *et al.*, 2020 ▸).
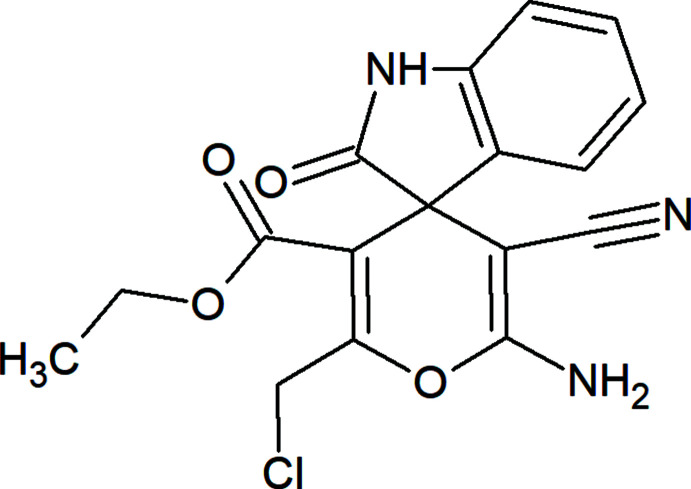


In this work, in the framework of our ongoing structural studies (Akkurt *et al.*, 2018 ▸; Naghiyev *et al.*, 2020 ▸, 2021 ▸), we report the crystal structure and Hirshfeld surface analysis of the title compound, ethyl 6′-amino-2′-(chloro­meth­yl)-5′-cyano-2-oxo-1,2-di­hydro­spiro­[indoline-3,4′-pyran]-3′-carb­oxy­l­ate.

## Structural commentary   

**Figure 2:**
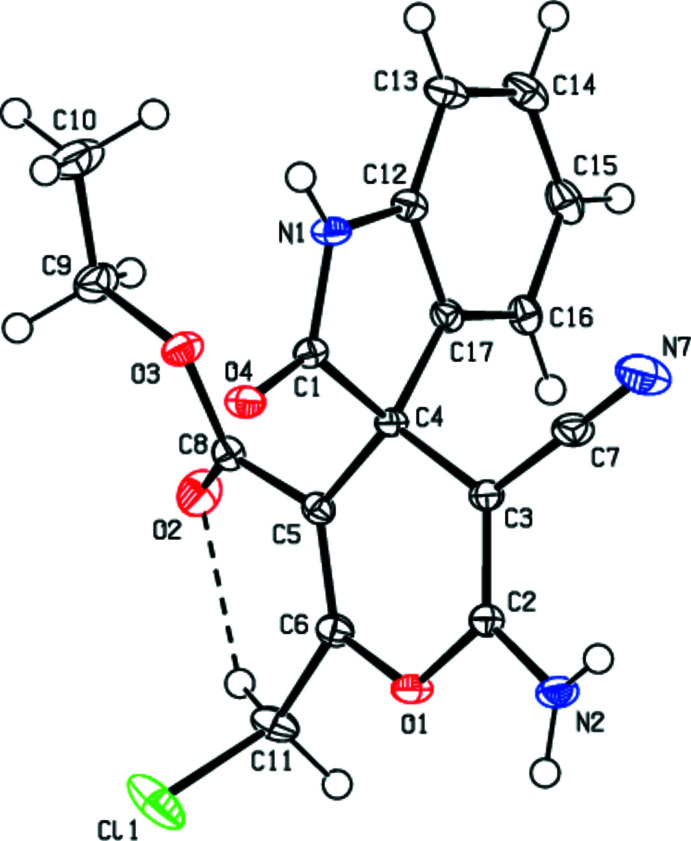
The mol­ecular structure of the title compound, showing the atom-numbering scheme and displacement ellipsoids at the 50% probability level.

In the title compound (Fig. 2 ▸), the 2,3-di­hydro-1*H*-indole ring system (N1/C1/C4/C12–C17) is nearly planar [maximum deviation = 0.039 (1) Å for C1], while the 4*H*-pyran ring (O1/C2–C6) adopts a flattened-boat conformation [puckering parameters (Cremer & Pople, 1975 ▸): *Q*_T_ = 0.1091 (13) Å, θ = 77.0 (6) ° and φ = 139.6 (7) °]. The planes of the 2,3-di­hydro-1*H*-indole ring system and the 4*H*-pyran ring are approximately perpendicular to each other, subtending a dihedral angle of 84.52 (5)°. The C5—C6—C11—Cl1, C6—C5—C8—O2, C6—C5—C8—O3, C5—C8—O3—C9 and C8—O3—C9—C10 torsion angles are −103.28 (13), −29.78 (18), 150.69 (11), 178.03 (10) and −169.29 (12)°, respectively. An intra­molecular C11—H11*B*⋯O2 contact stabilizes the mol­ecular conformation of the title compound (Fig. 2 ▸, Table 1 ▸), generating an *S*(6) ring motif (Bernstein *et al.*, 1995 ▸).

## Supra­molecular features   

**Figure 3:**
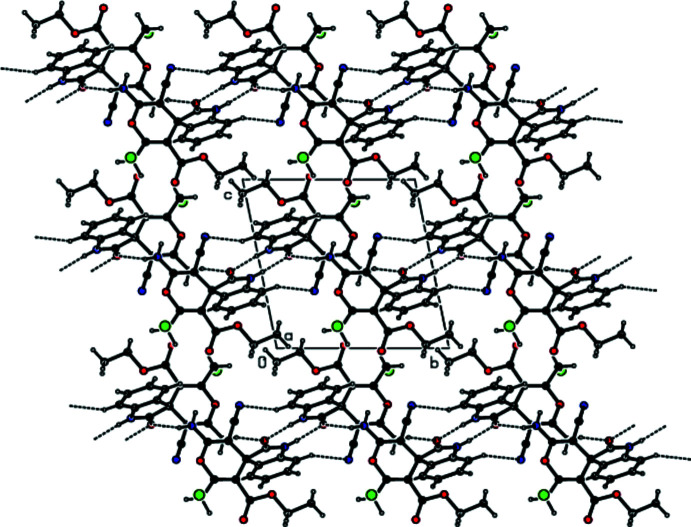
A view of the inter­molecular N—H⋯O and C—H⋯N hydrogen bonds in the crystal packing of the title compound down the *a* axis.

In the crystal, the mol­ecules are joined by N—H⋯O hydrogen-bond pairs along the *b*-axis direction as dimers with 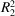
(8) and 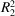
(14) ring motifs and by inter­molecular C—H⋯N hydrogen bonds as ribbons (Table 1 ▸; Figs. 3 ▸ and 4 ▸). Between the ribbons are only weak van der Waals contacts (Table 2 ▸). There are no C—H⋯π or π–π inter­actions in the crystal structure.

## Hirshfeld surface analysis   

**Figure 5:**
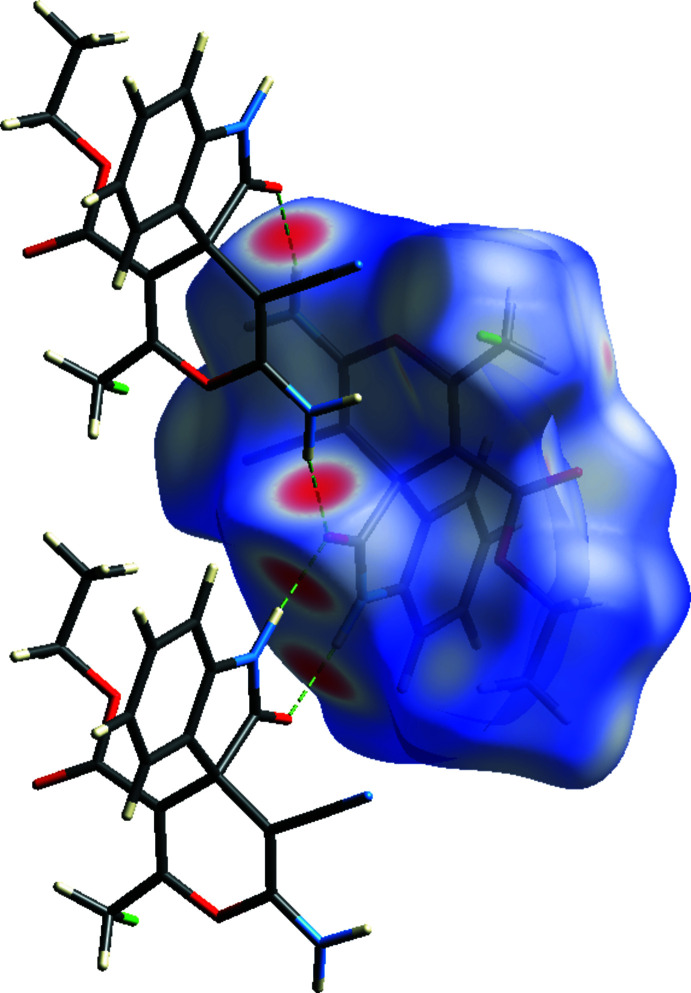
Hirshfeld surface of the title compound mapped with *d*_norm_ in the range −0.6053 to 1.4079 a.u.

**Figure 6:**
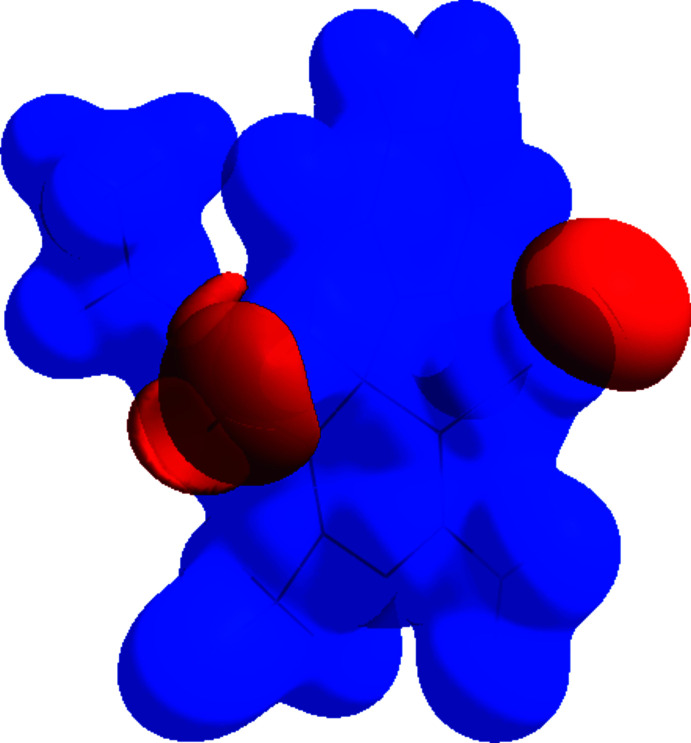
View of the three-dimensional Hirshfeld surface of the title compound plotted over electrostatic potential energy in the range −0.0500 to 0.0500 a.u. using the STO-3 G basis set at the Hartree–Fock level of theory. Hydrogen-bond donors and acceptors are shown as blue and red regions around the atoms, corresponding to positive and negative potentials, respectively.

A Hirshfeld surface analysis was performed to investigate the inter­molecular inter­actions (Tables 1 ▸ and 2 ▸) qu­anti­tatively and the associated two-dimensional fingerprint plots (McKinnon *et al.*, 2007 ▸) were generated with *CrystalExplorer17* (Turner *et al.*, 2017 ▸). The Hirshfeld surface plotted over *d*_norm_ in the range −0.6053 to 1.4079 a.u. is shown in Fig. 5 ▸. The red spots on the Hirshfeld surface represent N—H⋯O contacts. The Hirshfeld surface mapped over electrostatic potential (Spackman *et al.*, 2008 ▸) is shown in Fig. 6 ▸. The positive electrostatic potential (blue region) over the surface indicates hydrogen-donor potential, whereas the hydrogen-bond acceptors are represented by negative electrostatic potential (red region).

**Figure 7:**
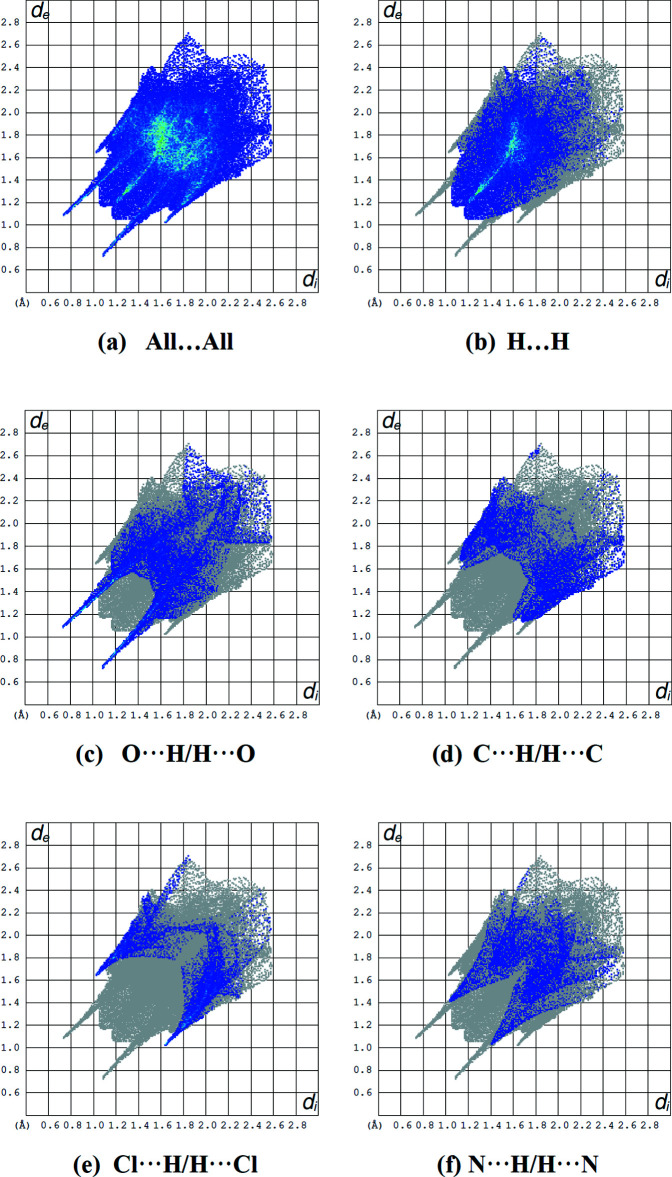
The two-dimensional fingerprint plots of the title compound, showing (*a*) all inter­actions, and those delineated into (*b*) H⋯H, (*c*) O⋯H/H⋯O, (*d*) C⋯H/H⋯C, (*e*) Cl⋯H/H⋯Cl and (*f*) N⋯H/H⋯N inter­actions [*d*_e_ and *d*_i_ represent the distances from a point on the Hirshfeld surface to the nearest atoms outside (external) and inside (inter­nal) the surface, respectively].

Fig. 7 ▸ shows the full two-dimensional fingerprint plot and those delineated into the major contacts: the H⋯H (34.9%; Fig. 7 ▸ *b*) inter­actions are the major factor in the crystal packing with O⋯H/H⋯O (19.2%; Fig. 7 ▸ *c*), C⋯H/H⋯C (11.9%; Fig. 7 ▸ *d*), Cl⋯H/H⋯Cl (10.7%; Fig. 7 ▸ *e*) and N⋯H/H⋯N (10.4%; Fig. 7 ▸ *f*) inter­actions representing the next highest contributions. Other weak inter­actions (contribution percent­ages) are O⋯N/N⋯O (2.3%), O⋯C/C⋯O (2.1%), N⋯C/C⋯N (2.1%), Cl⋯N/N⋯Cl (1.7%), Cl⋯O/O⋯Cl (1.4%), C⋯C (1.0%), N⋯N (0.7%), O⋯O (0.6%), Cl⋯C/C⋯Cl (0.6%) and Cl⋯Cl (0.3%).

## Database survey   

A survey of the Cambridge Structural Database (CSD version 5.41, update of March 2020; Groom *et al.*, 2016 ▸) using 2-amino-6-(chloro­meth­yl)-4*H*-pyran-3-carbo­nitrile as the main skeleton revealed the presence of three structures, ethyl 6-amino-2-(chloro­meth­yl)-5-cyano-4-(*o*-tol­yl)-4*H*-pyran-3-carb­oxyl­ate (CSD refcode HIRNUS; Athimoolam *et al.*, 2007 ▸), 2-amino-6-chloro­methyl-3-cyano-5-eth­oxy­carbonyl-4-(2-fur­yl)-4*H*-pyran (JEGWEX; Lokaj *et al.*, 1990 ▸) and ethyl 6′-amino-2′-(chloro­meth­yl)-5′-cyano-2-oxo-1,2-di­hydro­spiro­[indole-3,4′-pyran]-3′-carboxyl­ate (WIMBEC; Magerramov *et al.*, 2018 ▸).

In the crystal of HIRNUS, the six-membered pyran ring adopts a near-boat conformation. The crystal structure features two intra­molecular C—H⋯O inter­actions and the crystal packing is stabilized by inter­molecular N—H⋯O hydrogen bonds. These lead to two primary motifs, *viz. R* ^2^_2_(12) and *C*(8). Combination of these primary motifs leads to a secondary 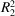
(20) ring motif.

In the crystal of JEGWEX, a potential precursor for fluoro­quinoline synthesis, the pyran ring is nearly planar, with the most outlying atoms displaced from the best-plane fit through all non-H atoms by 0.163 (2) and 0.118 (2) Å. The mol­ecules are arranged in layers oriented parallel to the (011) plane. In addition, the mol­ecules are linked by a weak C—H⋯O hydrogen bond, which gives rise to chains with base vector [111].

In WIMBEC, the pyran ring exhibits a near-boat conformation with puckering parameters *Q*_T_ = 0.085 (7) Å, θ = 84 (5)° and φ = 154 (5)°. In the crystal, mol­ecules are linked as dimers by pairs of N—H⋯O hydrogen bonds, forming ribbons along the *b*-axis direction. These ribbons are connected by weak van der Waals inter­actions, stabilizing the mol­ecular packing.

## Synthesis and crystallization   

The title compound was synthesized using previously reported procedures (Luo *et al.*, 2015 ▸; Magerramov *et al.*, 2018 ▸), and colourless needles were obtained upon recrystallization from methanol solution.

## Refinement details   

Crystal data, data collection and structure refinement details are summarized in Table 3 ▸. The H atoms of the NH and NH_2_ groups were located in a difference map, and their positional parameters were allowed to freely refine [N1—H1 = 0.853 (17), N2—H2*A* = 0.843 (19) and N2—H2*B* = 0.889 (18) Å], but their isotropic displacement parameters were constrained to take a value of 1.2*U*_eq_(N). All H atoms bound to C atoms were positioned geometrically and refined as riding with C—H = 0.95 (aromatic), 0.99 (methyl­ene) and 0.98 Å (meth­yl), with *U*_iso_(H) = 1.5*U*_eq_(C) for methyl H atoms and 1.2*U*_eq_(C) for the others. Four reflections, 0 0 1, 0 1 0, 1 0 0 and 1 2 0, affected by the incident beam-stop and owing to poor agreement between observed and calculated intensities, and five outliers, 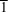 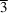 3, 3 1 1, 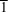 1 4, 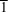 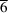 9 and 4 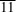 2, were omitted in the final cycles of refinement.

## Supplementary Material

Crystal structure: contains datablock(s) I. DOI: 10.1107/S2056989021006459/vm2250sup1.cif

Structure factors: contains datablock(s) I. DOI: 10.1107/S2056989021006459/vm2250Isup2.hkl

Supporting information file. DOI: 10.1107/S2056989021006459/vm2250Isup3.cml

CCDC reference: 2091350

Additional supporting information:  crystallographic information; 3D view; checkCIF report

## Figures and Tables

**Figure 4:**
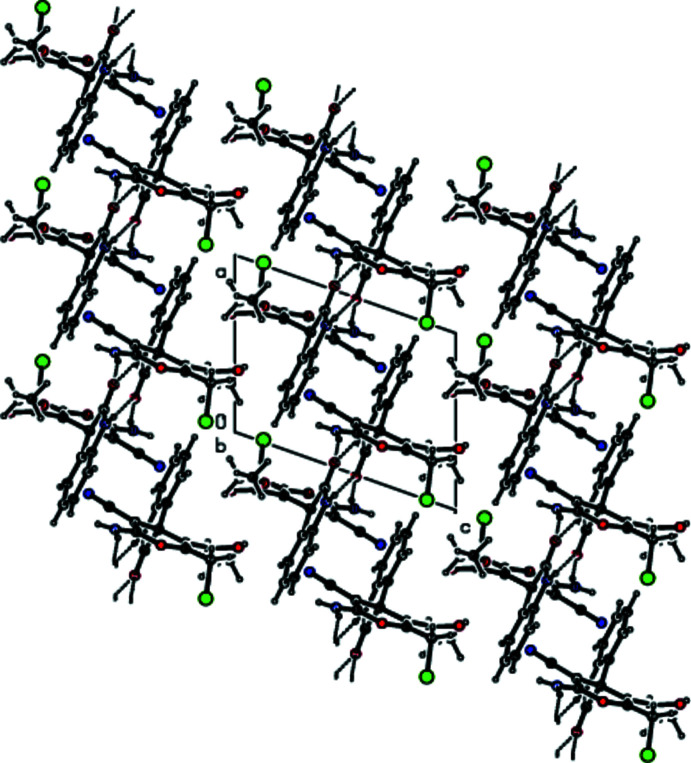
A view of the inter­molecular N—H⋯O and C—H⋯N hydrogen bonds in the crystal packing of the title compound down the *b* axis.

**Table 1:** Hydrogen-bond geometry (Å, °)

*D*—H⋯*A*	*D*—H	H⋯*A*	*D*⋯*A*	*D*—H⋯*A*
N1—H1⋯O4^i^	0.853 (17)	1.981 (17)	2.8292 (14)	173.0 (17)
N2—H2*B*⋯O4^ii^	0.887 (18)	2.095 (18)	2.9636 (15)	166.0 (17)
C11—H11*B*⋯O2	0.99	2.15	2.9039 (17)	131
C13—H13⋯N7^iii^	0.95	2.56	3.333 (2)	138

Symmetry codes: (i) -x, -y, -z+1; (ii) -x, -y+1, -z+1; (iii) -x+1, -y, -z+1.

**Table 2:** Summary of short inter­atomic contacts (Å) in the title compound

Contact	Distance	Symmetry operation
O3⋯H15	2.88	−1 + *x*, *y*, *z*
H9*A*⋯Cl1	3.06	−*x*, 1 − *y*, 2 − *z*
H2*B*⋯O4	2.095	−*x*, 1 − *y*, 1 − *z*
H16⋯H11*B*	2.37	1 − *x*, 1 − *y*, 2 − *z*
H1⋯O4	1.981	−*x*, −*y*, 1 − *z*
H16⋯H2*A*	2.49	1 − *x*, 1 − *y*, 1 − *z*
H13⋯N7	2.56	1 − *x*, −*y*, 1 − *z*
H10*A*⋯H11*A*	2.49	*x*, − 1 + *y*, *z*
H10*A*⋯C14	2.93	1 − *x*, −*y*, 2 − *z*

**Table 3:** Experimental details

Crystal data	
Chemical formula	C_17_H_14_ClN_3_O_4_
*M*_r_	359.76
Crystal system, space group	Triclinic, *P*\overline{1}
Temperature (K)	100
*a*, *b*, *c* (Å)	8.0218 (2), 10.2278 (3), 10.6714 (3)
α, β, γ (°)	98.8503 (7), 108.0048 (7), 96.3852 (6)
*V* (Å^3^)	810.92 (4)
*Z*	2
Radiation type	Mo *K*α
μ (mm^−1^)	0.26
Crystal size (mm)	0.25 × 0.20 × 0.15

Data collection	
Diffractometer	Bruker D8 QUEST PHOTON-III CCD
Absorption correction	Multi-scan (*SADABS*; Krause *et al.*, 2015 ▸)
*T*_min_, *T*_max_	0.903, 0.949
No. of measured, independent and observed [*I* > 2σ(*I*)] reflections	18865, 5899, 4685
*R*_int_	0.039
(sin θ/λ)_max_ (Å^−1^)	0.758

Refinement	
*R*[*F*^2^ > 2σ(*F* ^2^)], *wR*(*F* ^2^), *S*	0.044, 0.106, 1.03
No. of reflections	5899
No. of parameters	236
H-atom treatment	H atoms treated by a mixture of independent and constrained refinement
Δρ_max_, Δρ_min_ (e Å^−3^)	0.44, −0.63

Computer programs: *APEX3* (Bruker, 2018 ▸), *SAINT* (Bruker, 2013 ▸), *SHELXT2014/5* (Sheldrick, 2015*a* ▸), *SHELXL2018/3* (Sheldrick, 2015*b* ▸), *ORTEP-3 for Windows* (Farrugia, 2012 ▸) and *PLATON* (Spek, 2020 ▸).

## supplementary crystallographic information

### Crystal data

**Table:**

C_17_H_14_ClN_3_O_4_	*Z* = 2
*M**_r_* = 359.76	*F*(000) = 372
Triclinic, *P*1	*D*_x_ = 1.473 Mg m^−^^3^
*a* = 8.0218 (2) Å	Mo *K*α radiation, λ = 0.71073 Å
*b* = 10.2278 (3) Å	Cell parameters from 6340 reflections
*c* = 10.6714 (3) Å	θ = 2.6–32.5°
α = 98.8503 (7)°	µ = 0.26 mm^−^^1^
β = 108.0048 (7)°	*T* = 100 K
γ = 96.3852 (6)°	Prism, colourless
*V* = 810.92 (4) Å^3^	0.25 × 0.20 × 0.15 mm

### Data collection

**Table:**

Bruker D8 QUEST PHOTON-III CCD diffractometer	4685 reflections with *I* > 2σ(*I*)
φ and ω scans	*R*_int_ = 0.039
Absorption correction: multi-scan (SADABS; Krause *et al.*, 2015)	θ_max_ = 32.6°, θ_min_ = 2.6°
*T*_min_ = 0.903, *T*_max_ = 0.949	*h* = −12→12
18865 measured reflections	*k* = −15→15
5899 independent reflections	*l* = −16→16

### Refinement

**Table:**

Refinement on *F*^2^	Primary atom site location: difference Fourier map
Least-squares matrix: full	Secondary atom site location: difference Fourier map
*R*[*F*^2^ > 2σ(*F*^2^)] = 0.044	Hydrogen site location: mixed
*wR*(*F*^2^) = 0.106	H atoms treated by a mixture of independent and constrained refinement
*S* = 1.03	*w* = 1/[σ^2^(*F*_o_^2^) + (0.0366*P*)^2^ + 0.4187*P*] where *P* = (*F*_o_^2^ + 2*F*_c_^2^)/3
5899 reflections	(Δ/σ)_max_ = 0.001
236 parameters	Δρ_max_ = 0.44 e Å^−^^3^
0 restraints	Δρ_min_ = −0.63 e Å^−^^3^

### Special details

**Table:**

Geometry. All esds (except the esd in the dihedral angle between two l.s. planes) are estimated using the full covariance matrix. The cell esds are taken into account individually in the estimation of esds in distances, angles and torsion angles; correlations between esds in cell parameters are only used when they are defined by crystal symmetry. An approximate (isotropic) treatment of cell esds is used for estimating esds involving l.s. planes.

### Fractional atomic coordinates and isotropic or equivalent isotropic displacement parameters (Å^2^)

**Table:**

	*x*	*y*	*z*	*U*_iso_*/*U*_eq_	
Cl1	−0.00786 (5)	0.62296 (4)	0.86942 (4)	0.03426 (11)	
O1	0.20564 (12)	0.57639 (8)	0.66693 (9)	0.01580 (17)	
O2	0.34827 (14)	0.39501 (10)	1.01602 (9)	0.0221 (2)	
O3	0.24269 (13)	0.20082 (9)	0.86649 (9)	0.01705 (17)	
O4	−0.01410 (11)	0.18103 (8)	0.54781 (9)	0.01560 (17)	
N1	0.22730 (14)	0.07160 (10)	0.58525 (11)	0.01426 (19)	
H1	0.171 (2)	−0.0075 (17)	0.5472 (17)	0.017*	
N2	0.20156 (16)	0.58493 (11)	0.45919 (12)	0.0189 (2)	
H2A	0.198 (2)	0.5484 (18)	0.3819 (19)	0.023*	
H2B	0.158 (2)	0.6601 (18)	0.4714 (18)	0.023*	
C1	0.14677 (15)	0.18025 (11)	0.59191 (11)	0.0124 (2)	
C2	0.23180 (15)	0.51172 (11)	0.55510 (12)	0.0137 (2)	
C3	0.28549 (15)	0.38996 (11)	0.54920 (12)	0.0131 (2)	
C4	0.29313 (15)	0.30748 (11)	0.65670 (11)	0.01141 (19)	
C5	0.27019 (15)	0.39250 (11)	0.77759 (11)	0.01218 (19)	
C6	0.23527 (15)	0.51749 (12)	0.77729 (12)	0.0138 (2)	
C7	0.32238 (18)	0.33266 (13)	0.43295 (13)	0.0182 (2)	
N7	0.3547 (2)	0.28501 (14)	0.33968 (13)	0.0300 (3)	
C8	0.29278 (15)	0.33459 (12)	0.90096 (12)	0.0139 (2)	
C9	0.2632 (2)	0.12998 (14)	0.97758 (14)	0.0231 (3)	
H9A	0.1751	0.1491	1.0225	0.028*	
H9B	0.3840	0.1588	1.0446	0.028*	
C10	0.2340 (3)	−0.01675 (15)	0.91875 (16)	0.0317 (3)	
H10A	0.2419	−0.0684	0.9899	0.048*	
H10B	0.3251	−0.0348	0.8779	0.048*	
H10C	0.1160	−0.0431	0.8500	0.048*	
C11	0.21988 (17)	0.61386 (13)	0.89102 (13)	0.0187 (2)	
H11A	0.2843	0.7039	0.8960	0.022*	
H11B	0.2752	0.5848	0.9765	0.022*	
C12	0.41277 (15)	0.10561 (12)	0.64735 (12)	0.0137 (2)	
C13	0.53735 (17)	0.02074 (13)	0.66518 (13)	0.0181 (2)	
H13	0.5037	−0.0737	0.6349	0.022*	
C14	0.71502 (17)	0.08061 (14)	0.72987 (13)	0.0201 (2)	
H14	0.8044	0.0255	0.7431	0.024*	
C15	0.76464 (16)	0.21894 (14)	0.77546 (13)	0.0188 (2)	
H15	0.8866	0.2568	0.8189	0.023*	
C16	0.63576 (16)	0.30242 (12)	0.75758 (12)	0.0150 (2)	
H16	0.6684	0.3968	0.7893	0.018*	
C17	0.45955 (15)	0.24390 (12)	0.69254 (11)	0.0126 (2)	

### Atomic displacement parameters (Å^2^)

**Table:**

	*U*^11^	*U*^22^	*U*^33^	*U*^12^	*U*^13^	*U*^23^
Cl1	0.01877 (16)	0.0398 (2)	0.0354 (2)	0.00475 (13)	0.00875 (14)	−0.01772 (16)
O1	0.0210 (4)	0.0116 (4)	0.0157 (4)	0.0066 (3)	0.0060 (3)	0.0028 (3)
O2	0.0293 (5)	0.0206 (4)	0.0140 (4)	−0.0004 (4)	0.0064 (4)	0.0013 (3)
O3	0.0244 (4)	0.0127 (4)	0.0150 (4)	0.0044 (3)	0.0067 (3)	0.0042 (3)
O4	0.0128 (4)	0.0121 (4)	0.0196 (4)	0.0027 (3)	0.0029 (3)	0.0014 (3)
N1	0.0146 (4)	0.0092 (4)	0.0171 (5)	0.0030 (3)	0.0032 (4)	0.0006 (3)
N2	0.0264 (6)	0.0154 (5)	0.0196 (5)	0.0099 (4)	0.0099 (4)	0.0080 (4)
C1	0.0139 (5)	0.0103 (5)	0.0128 (5)	0.0022 (4)	0.0041 (4)	0.0020 (4)
C2	0.0137 (5)	0.0124 (5)	0.0157 (5)	0.0032 (4)	0.0052 (4)	0.0033 (4)
C3	0.0152 (5)	0.0114 (5)	0.0137 (5)	0.0037 (4)	0.0055 (4)	0.0030 (4)
C4	0.0125 (5)	0.0091 (4)	0.0129 (5)	0.0029 (4)	0.0044 (4)	0.0021 (4)
C5	0.0114 (5)	0.0124 (5)	0.0121 (5)	0.0021 (4)	0.0037 (4)	0.0009 (4)
C6	0.0136 (5)	0.0130 (5)	0.0135 (5)	0.0029 (4)	0.0032 (4)	0.0010 (4)
C7	0.0233 (6)	0.0174 (5)	0.0178 (6)	0.0101 (5)	0.0079 (5)	0.0079 (4)
N7	0.0461 (8)	0.0317 (6)	0.0223 (6)	0.0239 (6)	0.0170 (6)	0.0113 (5)
C8	0.0127 (5)	0.0144 (5)	0.0156 (5)	0.0028 (4)	0.0059 (4)	0.0029 (4)
C9	0.0362 (7)	0.0196 (6)	0.0195 (6)	0.0091 (5)	0.0134 (5)	0.0097 (5)
C10	0.0528 (10)	0.0182 (6)	0.0293 (7)	0.0106 (6)	0.0163 (7)	0.0111 (6)
C11	0.0182 (5)	0.0175 (5)	0.0169 (5)	0.0065 (4)	0.0029 (4)	−0.0032 (4)
C12	0.0142 (5)	0.0137 (5)	0.0136 (5)	0.0047 (4)	0.0045 (4)	0.0027 (4)
C13	0.0220 (6)	0.0171 (5)	0.0173 (6)	0.0106 (4)	0.0071 (5)	0.0035 (4)
C14	0.0194 (6)	0.0270 (6)	0.0171 (6)	0.0131 (5)	0.0066 (5)	0.0056 (5)
C15	0.0130 (5)	0.0288 (6)	0.0157 (5)	0.0065 (4)	0.0049 (4)	0.0049 (5)
C16	0.0145 (5)	0.0185 (5)	0.0128 (5)	0.0030 (4)	0.0056 (4)	0.0033 (4)
C17	0.0136 (5)	0.0144 (5)	0.0111 (5)	0.0041 (4)	0.0050 (4)	0.0027 (4)

### Geometric parameters (Å, º)

**Table:**

Cl1—C11	1.7842 (13)	C6—C11	1.4871 (17)
O1—C2	1.3595 (15)	C7—N7	1.1550 (18)
O1—C6	1.3725 (14)	C9—C10	1.497 (2)
O2—C8	1.2063 (15)	C9—H9A	0.9900
O3—C8	1.3417 (14)	C9—H9B	0.9900
O3—C9	1.4586 (15)	C10—H10A	0.9800
O4—C1	1.2308 (14)	C10—H10B	0.9800
N1—C1	1.3495 (14)	C10—H10C	0.9800
N1—C12	1.4064 (15)	C11—H11A	0.9900
N1—H1	0.853 (17)	C11—H11B	0.9900
N2—C2	1.3379 (15)	C12—C13	1.3837 (16)
N2—H2A	0.843 (19)	C12—C17	1.3907 (16)
N2—H2B	0.889 (18)	C13—C14	1.3966 (19)
C1—C4	1.5592 (16)	C13—H13	0.9500
C2—C3	1.3610 (15)	C14—C15	1.393 (2)
C3—C7	1.4183 (17)	C14—H14	0.9500
C3—C4	1.5156 (16)	C15—C16	1.3995 (17)
C4—C5	1.5138 (16)	C15—H15	0.9500
C4—C17	1.5183 (16)	C16—C17	1.3840 (16)
C5—C6	1.3390 (16)	C16—H16	0.9500
C5—C8	1.4944 (16)		
			
C2—O1—C6	119.01 (9)	C10—C9—H9A	110.3
C8—O3—C9	115.95 (10)	O3—C9—H9B	110.3
C1—N1—C12	111.77 (10)	C10—C9—H9B	110.3
C1—N1—H1	123.4 (11)	H9A—C9—H9B	108.6
C12—N1—H1	124.8 (11)	C9—C10—H10A	109.5
C2—N2—H2A	118.0 (12)	C9—C10—H10B	109.5
C2—N2—H2B	119.3 (11)	H10A—C10—H10B	109.5
H2A—N2—H2B	120.6 (16)	C9—C10—H10C	109.5
O4—C1—N1	126.32 (11)	H10A—C10—H10C	109.5
O4—C1—C4	125.13 (10)	H10B—C10—H10C	109.5
N1—C1—C4	108.42 (9)	C6—C11—Cl1	110.59 (9)
N2—C2—O1	110.94 (10)	C6—C11—H11A	109.5
N2—C2—C3	127.13 (11)	Cl1—C11—H11A	109.5
O1—C2—C3	121.91 (10)	C6—C11—H11B	109.5
C2—C3—C7	118.87 (11)	Cl1—C11—H11B	109.5
C2—C3—C4	122.67 (10)	H11A—C11—H11B	108.1
C7—C3—C4	118.26 (10)	C13—C12—C17	122.36 (11)
C5—C4—C3	109.52 (9)	C13—C12—N1	128.15 (11)
C5—C4—C17	112.86 (9)	C17—C12—N1	109.49 (10)
C3—C4—C17	112.55 (9)	C12—C13—C14	116.79 (12)
C5—C4—C1	113.49 (9)	C12—C13—H13	121.6
C3—C4—C1	107.25 (9)	C14—C13—H13	121.6
C17—C4—C1	100.85 (9)	C15—C14—C13	121.72 (11)
C6—C5—C8	120.03 (10)	C15—C14—H14	119.1
C6—C5—C4	121.88 (10)	C13—C14—H14	119.1
C8—C5—C4	118.07 (9)	C14—C15—C16	120.36 (12)
C5—C6—O1	123.81 (11)	C14—C15—H15	119.8
C5—C6—C11	127.27 (11)	C16—C15—H15	119.8
O1—C6—C11	108.91 (10)	C17—C16—C15	118.24 (11)
N7—C7—C3	178.80 (14)	C17—C16—H16	120.9
O2—C8—O3	123.11 (11)	C15—C16—H16	120.9
O2—C8—C5	127.00 (11)	C16—C17—C12	120.53 (11)
O3—C8—C5	109.89 (10)	C16—C17—C4	130.24 (11)
O3—C9—C10	106.89 (11)	C12—C17—C4	109.23 (10)
O3—C9—H9A	110.3		
			
C12—N1—C1—O4	−179.07 (12)	C2—O1—C6—C5	−7.59 (17)
C12—N1—C1—C4	4.71 (13)	C2—O1—C6—C11	173.03 (10)
C6—O1—C2—N2	−178.30 (10)	C9—O3—C8—O2	−1.51 (17)
C6—O1—C2—C3	0.39 (17)	C9—O3—C8—C5	178.03 (10)
N2—C2—C3—C7	2.90 (19)	C6—C5—C8—O2	−29.78 (18)
O1—C2—C3—C7	−175.56 (11)	C4—C5—C8—O2	148.52 (12)
N2—C2—C3—C4	−171.94 (12)	C6—C5—C8—O3	150.69 (11)
O1—C2—C3—C4	9.61 (18)	C4—C5—C8—O3	−31.00 (14)
C2—C3—C4—C5	−11.44 (15)	C8—O3—C9—C10	−169.29 (12)
C7—C3—C4—C5	173.69 (10)	C5—C6—C11—Cl1	−103.28 (13)
C2—C3—C4—C17	−137.85 (12)	O1—C6—C11—Cl1	76.07 (11)
C7—C3—C4—C17	47.28 (14)	C1—N1—C12—C13	176.98 (12)
C2—C3—C4—C1	112.13 (12)	C1—N1—C12—C17	−2.67 (14)
C7—C3—C4—C1	−62.74 (13)	C17—C12—C13—C14	−0.81 (18)
O4—C1—C4—C5	58.07 (15)	N1—C12—C13—C14	179.59 (12)
N1—C1—C4—C5	−125.66 (10)	C12—C13—C14—C15	0.58 (19)
O4—C1—C4—C3	−63.02 (15)	C13—C14—C15—C16	0.2 (2)
N1—C1—C4—C3	113.24 (10)	C14—C15—C16—C17	−0.69 (18)
O4—C1—C4—C17	179.05 (11)	C15—C16—C17—C12	0.48 (17)
N1—C1—C4—C17	−4.69 (12)	C15—C16—C17—C4	−178.77 (11)
C3—C4—C5—C6	4.62 (15)	C13—C12—C17—C16	0.29 (18)
C17—C4—C5—C6	130.85 (11)	N1—C12—C17—C16	179.96 (10)
C1—C4—C5—C6	−115.19 (12)	C13—C12—C17—C4	179.68 (11)
C3—C4—C5—C8	−173.65 (9)	N1—C12—C17—C4	−0.65 (13)
C17—C4—C5—C8	−47.42 (13)	C5—C4—C17—C16	−56.14 (16)
C1—C4—C5—C8	66.54 (13)	C3—C4—C17—C16	68.46 (15)
C8—C5—C6—O1	−177.31 (10)	C1—C4—C17—C16	−177.56 (12)
C4—C5—C6—O1	4.46 (18)	C5—C4—C17—C12	124.55 (10)
C8—C5—C6—C11	1.96 (18)	C3—C4—C17—C12	−110.86 (11)
C4—C5—C6—C11	−176.28 (11)	C1—C4—C17—C12	3.13 (12)

### Hydrogen-bond geometry (Å, º)

**Table:**

*D*—H···*A*	*D*—H	H···*A*	*D*···*A*	*D*—H···*A*
N1—H1···O4^i^	0.853 (17)	1.981 (17)	2.8292 (14)	173.0 (17)
N2—H2*B*···O4^ii^	0.887 (18)	2.095 (18)	2.9636 (15)	166.0 (17)
C11—H11*B*···O2	0.99	2.15	2.9039 (17)	131
C13—H13···N7^iii^	0.95	2.56	3.333 (2)	138

Symmetry codes: (i) −*x*, −*y*, −*z*+1; (ii) −*x*, −*y*+1, −*z*+1; (iii) −*x*+1, −*y*, −*z*+1.
